# Correction: Effect of heterocycle content on metal binding isostere coordination

**DOI:** 10.1039/d2sc90048c

**Published:** 2022-03-08

**Authors:** Benjamin L. Dick, Ashay Patel, Seth M. Cohen

**Affiliations:** Department of Chemistry and Biochemistry, University of California San Diego 9500 Gilman Drive La Jolla CA 92093-0358 USA scohen@ucsd.edu

## Abstract

Correction for ‘Effect of heterocycle content on metal binding isostere coordination’ by Benjamin L. Dick *et al.*, *Chem. Sci.*, 2020, **11**, 6907–6914, DOI: 10.1039/D0SC02717K.

The authors regret that a complete Conflicts of interest section was not shown in the original article. The correct Conflicts of interest section is shown below.

## Conflicts of interest

The authors declare the following competing financial interest(s): S. M. C. is a co-founder of and has an equity interest in Cleave Therapeutics, Forge Therapeutics, and Blacksmith Medicines, companies that may potentially benefit from the research results. S. M. C. also serves on the Scientific Advisory Board for Blacksmith Medicines and serves on the Scientific Advisory Board and receives compensation from Forge Therapeutics. The terms of this arrangement have been reviewed and approved by the University of California, San Diego in accordance with its conflict of interest policies.

The Royal Society of Chemistry apologises for these errors and any consequent inconvenience to authors and readers.

## Supplementary Material

